# Female Medical Students

**Published:** 1848-02

**Authors:** 


*Female Medical Students.*—As was to have been expected, the announcement of a female pursuing a regular course of medical education, excites not a little remark in private circles, and the public prints. We perceive an article going the rounds of the newspapers which states that a female medical student is in attendance at Boston, Mass. We presume this is an error, as the Boston school was one of the number, admission to which was denied to the person referred to in the article contained in our last number. There is some difference of opinion in the minds of medical men as regards the propriety of the course pursued by Geneva College in the matter, but we are not aware that any of the parties immediately concerned are dissatisfied. If any doubt the ability of females to become scientific practitioners in certain departments of our art, we might refer to Madame Boivin, who is not only an accomplished accoucheur, but eminent as a teacher and writer. Will it ever be incumbent on the medical teacher to say *ladies and gentlemen*, instead of *gentlemen? Nous verrons.*

